# Cesarean Scar Pregnancy Successfully Managed to Term: When the Patient Is Determined to Keep the Pregnancy

**DOI:** 10.3390/medicina56100496

**Published:** 2020-09-24

**Authors:** Ranko Kutlesic, Marija Kutlesic, Predrag Vukomanovic, Milan Stefanovic, Danka Mostic-Stanisic

**Affiliations:** 1 Clinic of Gynaecology and Obstetrics, University Clinical Centre Nis, 18000 Nis, Serbia; predragvukomanovic@yahoo.com (P.V.); milstef64@gmail.com (M.S.); 2 Faculty of Medicine, University of Nis, 18000 Nis, Serbia; 3 Department of Anaesthesia, Clinic of Gynaecology and Obstetrics, University Clinical Centre Nis, 18000 Nis, Serbia; mkutlesic5@gmail.com; 4 Institute of Gynaecology and Obstetrics Belgrade, Clinical centre of Serbia, 11000 Belgrade, Serbia; tmostic@sbb.rs

**Keywords:** pregnancy, High-Risk, ectopic, cesarean section, complication

## Abstract

Cesarean scar pregnancy (CSP) is a rare form of ectopic pregnancy, defined as the implantation of the gestational sac at the uterine incision scar of the previous cesarean section. This condition is associated with severe maternal and fetal/neonatal complications, including severe bleeding, rupture of the uterus, fetal demise, or preterm delivery. In view of these, early diagnosis allows the option of termination of pregnancy. In this case report, we present a patient with a cesarean scar pregnancy who was diagnosed at the sixth week of gestation but declined early termination of the pregnancy and was managed to the 38th week. Placenta previa was confirmed in the second trimester. A planned cesarean section was performed that resulted in the birth of a live full-term neonate. Intraoperatively, placenta percreta was diagnosed, and due to uncontrollable bleeding, a hysterectomy was performed. The postoperative course was uneventful. In cases where an early diagnosis of CSP is made, women should be counseled that this will almost certainly evolve to placenta previa, and the associated risks should be explained. Close follow-up of CSP is mandatory if expectant management is selected. Further studies are needed for definitive conclusions and to determine the risks of expectant management.

## 1. Introduction

Cesarean scar pregnancy (CSP) is a rare form of ectopic pregnancy. CSP is a long-term complication of cesarean delivery, defined as the implantation of the gestational sac at the uterine incision scar of the previous cesarean section. Implantation of the pregnancy on or in a cesarean section scar is a precursor of placenta accreta spectrum disorders, including: Placenta accreta (villi are directly attached to the myometrium without interposing decidua), placenta increta (the villi penetrate the myometrium up to the uterine serosa) and placenta percreta (the villi penetrate through serosa and invade surrounding tissues and organs, such as the bladder) [1].

Today, the main cause for this complex obstetric complication is uterine surgery, in particular, uterine scar secondary to cesarean delivery [2].

Although placenta previa and placenta accreta have been known for many years, it seems that their connections with CSP have been established in the last fifteen years.

The prevalence of CSP is reported to be 1 in 800 to 2500 of all cesarean deliveries performed [3,4,5], with an increasing tendency, due to the increasing number of cesarean deliveries and better resolution of sonograms [6].

This condition is associated with severe maternal and fetal/neonatal complications, including severe bleeding, rupture of the uterus, fetal demise [6], and preterm delivery [7]. In this case report, we present a patient with a cesarean scar pregnancy, diagnosed at the sixth week of gestation, but declined early termination of the pregnancy and was managed to the 38th week.

## 2. Case Report

A 39-year-old (gravida 3, para 1) was admitted into our clinic, due to a 6-week history of amenorrhea, lower abdominal pain, and brown-colored vaginal discharge. Five years ago, the patient had a term cesarean delivery followed by an uneventful postoperative course and was otherwise healthy. The patient had no history of pelvic inflammatory disease or the use of intrauterine devices. Her menarche occurred at twelve years of age, and her menstrual cycles are regular.

**Figure 1:**
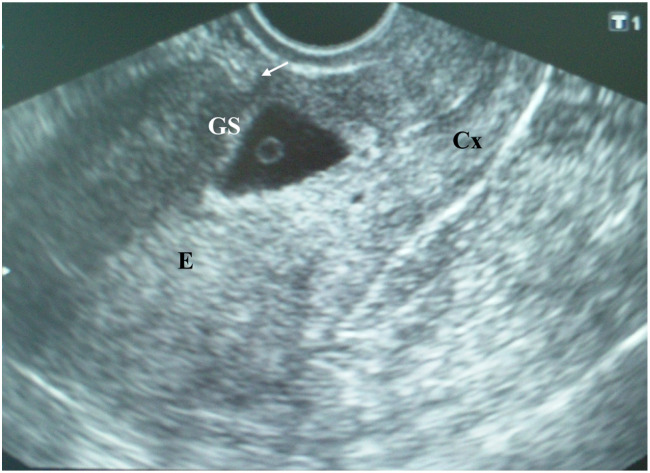
Transvaginal ultrasound examination of a cesarean scar pregnancy (CSP) at the sixth postmenstrual week showing an empty uterine cavity with a 9 mm endometrial strip (E) and a triangular gestational sac (10 mm in diameter) located within the isthmic part of the anterior uterine wall filling the niche of the scar, with a yolk sac inside covered with a thin myometrial layer 2 mm in diameter (arrow); the cervical channel (Cx) is empty; according to the presence of cross over sign (COS), this gestational sac could be identified as COS-1; according to the implantation of the gestation sac it is implanted in the niche of the scar (ultrasound sign reported by Kaelin Agten et al.); according to the position of the center of the gestational sac it could be classified as implantation bellow the uterine midline (classification proposed by Timor-Tritsch et al.) (explanation in *Discussion*).

On admission, the patient was hemodynamically stable. An examination of her cardiac and respiratory systems was unremarkable. Her abdomen was soft without tenderness. A speculum examination indicated the presence of a single cervix with brown-colored discharge from the external os and no other pathological findings. Bimanual pelvic examination revealed an enlarged soft uterus corresponding to the sixth gestational week; the patient’s cervix was closed with no pathological adnexal findings. Transvaginal ultrasound examination (TVUS) [Toshiba Nemio XG, 6 MHz] showed an empty uterine cavity with a 9 mm endometrial strip and a triangular gestational sac (10 mm in diameter) located within the isthmic part of the anterior uterine wall that filled the niche of the scar, with a yolk sac inside (Figure 1). Both ovaries appeared sonographically normal, with a corpus luteum on the left ovary. There was no intraperitoneal fluid in the pouch of Douglas. A color Doppler ultrasound image of the cesarean scar gestational sac showed peripheral hypervascularity.

**Figure 2:**
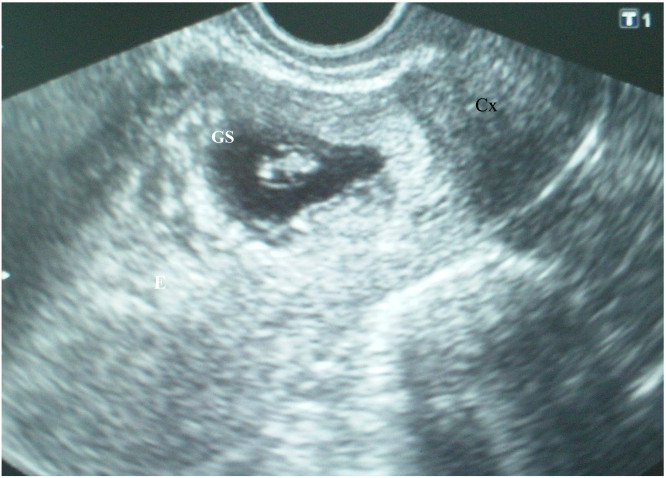
Cesarean scar pregnancy: A gestational sac (GS) containing an embryo with heart action and a crown–rump length (CRL) of 9.6 mm, which corresponded to 7 weeks and 1 day of gestation.

The patient’s laboratory results were within normal limits. Cesarean scar ectopic gravidity was suspected, and the patient was informed of the evolution to placenta previa, possibly to accreta, as well as of the associated risks. She was offered the option of termination. The patient, however, refused the termination of the pregnancy. Six days later, the patient was asymptomatic, and her abdominal pain disappeared, TVUS revealed a gestational sac containing an embryo with heart action and a crown–rump length (CRL) of 9.6 mm, which corresponded to 7 weeks and 1 day of gestation (Figure 2).

Once again, the patient and her family were informed about the situation, and the patient again refused termination of pregnancy, but accepted further close follow-up. Four weeks later, the ultrasound examination showed that the gestational sac was developing toward the uterine cavity, thereby allowing normal development of the embryo. Ultrasound examination at 22 weeks showed no evidence of fetal structural defects, and the low-lying placenta was found. Ultrasound examination performed at 26 weeks revealed an anterior placenta previa and sonographic features suggestive of a placenta accreta: Thin, deficient lower uterine segment with the decidual interface between the placenta and the myometrium, and large dilated blood vessels in the area.

The pregnancy developed uneventfully until the 38th gestational week when the planned cesarean section was performed. In the operating room, our patient was placed supine, and standard monitoring was initiated (noninvasive blood pressure, electrocardiography, pulse oxymetry, capnography, using bed-side monitor model BSM-230lk Nihon Kohden Corporation, Tokyo, Japan). Since placenta accreta was suspected and a heavy blood loss expected, we established two large-bore iv lines (16 G), and immediately started 500 mL saline with Ceftriaxone 2 g, as well as Ringer lactate solution 500 mL. In discussion with our patient, we chose to perform spinal anesthesia, but she accepted the possibility to combine it with general anesthesia in case of possible complications. In a right lateral position, 2.5 mL 0.5% Bupivacaine with 0.3 mL Fentanyl was injected in L3–L4 space. The patient was again placed supine with left uterine displacement, and phenylephrine infusion of 0.3–0.5 mcg/kg/min immediately started to prevent excessive spinal anesthesia-induced hypotension. Her initial BP was 125/65 mmHg, HR 90/min. Induction-delivery time lasted 15 min, during which BP was 90–100/50–60 mmHg, HR 90/min.

A lower transversal abdominal incision was performed, showing placental tissue that had invaded the parietal peritoneum and isthmic part of the uterus and protruded outside the uterus. A healthy female neonate (2420 g/48 cm, with Apgar scores of 8/9 at 1, 5 min, respectively) was extracted directly through placental tissue. The umbilical cord was clamped immediately after the delivery to avoid excessive fetal blood loss.

After the delivery of the neonate, massive bleeding started with BP drop to 70/35 mmHg, and it was decided to perform a hysterectomy. The patient was induced to general anesthesia with propofol 120 mg plus succinylcholine 100 mg. After the intubation, anesthesia was maintained with 0.8–1 vol% end-tidal sevoflurane and 50% nitrous oxide in oxygen. The lungs were mechanically ventilated to maintain end-tidal PCO2 of 28–32 mmHg, with a fresh gas flow of 6l/min.

The placenta had invaded the isthmic part of the uterus and the parietal peritoneum, and it was impossible to remove from the uterus (placenta percreta). Due to massive bleeding from the placental site, hysterectomy was performed. The estimated blood loss during the surgery was approximately 2500 mL. The operation was otherwise uncomplicated. The bladder was examined by a urologist consultant intraoperatively and was found not to be damaged during the surgery.

After the initial 1000 mL of crystalloid solution, aggressive iv fluid load continued. During the next 90 min of the operation and the first two postoperative hours, she received an additional 3000 mL of crystalloids, 500 mL Hydroxyethyl starch solution, 1050 mL red blood cell transfusion, 440 mL of fresh frozen plasma, 600 mL cryoprecipitate, 10 mL Calcium gluconate, 1 g tranexamic acid iv bolus during 10 min followed by 1mg/kg/h infusion. During the first 30 min after the delivery of the neonate, she was hypotensive—BP 75–80/40 mmHg, HR 80/min. During the next 60 min of the operation, the BP was rather satisfactory, 20–30% below baseline values, 90/55 mmHg, HR 80/min, and even reached 110/60 mmHg with HR 90/min at the end of the operation. After the extubation, the patient was conscious, breathed adequately (RR 11–12/min, SpO2 95%), and she was hemodynamically stable. Her immediate postoperative course passed uneventfully; she remained hemodynamically stable, with adequate diuresis and laboratory findings within the normal range, so there was no need for further blood product supplementation.

Further postoperative recovery was also uneventful, and 10 days later, the patient left the hospital, but with a healthy baby.

## 3. Discussion

CSP is a consequence of altered trophoblastic invasion in the place of a uterine cesarean scar in a subsequent pregnancy. In a normal pregnancy, the trophoblastic invasion is stopped by the decidua basalis, where a zone of fibrinoid degeneration is created, described as the Rohr stria and Nitabuch layer. At the area of the uterine cesarean scar, there is often an absence or partial disruption of the decidua basalis. Thus, the pregnancy is not adequately implanted in the decidualized endometrium, but rather embeds in the fibrous scar tissue and myometrium [8,9].

Trophoblast and villous tissue can invade deeply within the myometrium, including the myometrial vessels, and can reach the surrounding pelvic organs. The unusual myometrial environment is probably the cause of the cellular changes observed in placenta accreta spectrum [2]. Hemodynamic effects of abnormally deep placentation and transformation of the radial and arcuate arteries are causes of placental ultrasound and histopathological features associated with placenta accreta spectrum, which are more pronounced with the deeper invasion [10].

Clinically, abnormal implantation could be partially over the thick fibrous scar. The pregnancy could even be located entirely outside the uterus, connected by a narrow fistula and bulging into a broad ligament or uterovesical fold. It was reported that the most common forms of CSP are pregnancies implanted entirely within the myometrial deficiency or only with the part of trophoblast extending into the defect in the myometrium [1]. Information on the serosal vascularity, uterine dehiscence, and extension of the accrete area are also important to increase the quality of histological sampling [11].

Cesarean scar pregnancies represent a challenge for every clinician, not only to diagnose, but also to treat. Standard diagnostic findings for the diagnosis of CSP are as follows [6]: (1) No gestational sac in the uterine cavity or cervical channel; (2) a placenta and/or gestational sac embedded in the hysterotomy scar (in the lower uterine segment); (3) the myometrial layer between the gestational sac and bladder being thin (from 1–3 mm to 5 mm) or absent; (4) ultrasound examination in early gestation revealing a triangular gestational sac that fills the niche of the scar; (5) the presence of an embryonic/fetal pole and/or a yolk sac with or without heart activity; (6) a high velocity and low obstruction of blood flow around the gestational sac on color Doppler flow imaging; and (7) positive human chorionic gonadotropin (HCG) in the blood. All the mentioned criteria were present in our patient.

Early first-trimester ultrasound images from 6–8 weeks’ gestation are very important to predict the evolution of CSP. The crossover sign (COS) seems to be very useful for such purposes [12]. 

As it was described, in a sagittal view of the uterus, a straight line is drawn connecting the internal cervical os and the uterine fundus through endometrium (endometrial line). The gestational sac is identified, and its superior-inferior (S–I) diameter is traced perpendicular to the endometrial line. CSP could be categorized according to the relationship between the endometrial line and S–I diameter of the gestational sac into two groups: COS-1, in which the gestational sac is implanted within the Cesarean scar, and at least two-thirds of the S–I diameter is above the endometrial line; and COS-2 in which the gestational sac is implanted within the Cesarean scar, and less than two-thirds of the S–I diameter is above the endometrial line. The latter group could be further divided into two categories according to the presence (COS-2+) or absence (COS-2–) of an intersection of the S–I diameter and the endometrial line [13]. CSP with COS-2– may represent a milder variant that does not fulfill completely the proposed ultrasound criteria for CSP. According to this categorization, the gestational sac of our case could be identified as COS-1. It was reported that the proportion of cases with placenta percreta was significantly higher in women with COS-1 than in those with COS-2 (83.3% vs. 42.9%) [13].

Another study reported that in patients with COS-1 the estimated blood loss during the surgery was significantly higher, and the mean operative time was longer, with more packed red blood cell units required during or after the operation. The rate of iatrogenic preterm birth at <34 weeks’ gestation was higher compared to pregnancies with COS-2 [12].

Recent retrospective analysis of prospectively collected data from women with placenta previa and at least one previous cesarean delivery or uterine surgery reported that early first-trimester (5–7 weeks’ gestation) sonographic assessment of pregnancies with previous cesarean delivery can predict the ultrasound stage of placenta accreta spectrum disorder [14]. Three sonographic markers for first-trimester assessment of CSP were analyzed: Already mentioned crossover sign (reported by Cali et al.), implantation of the gestational sac on the scar vs. in the niche of the cesarean scar (reported by Kaelin Agten et al.), and position of the center of the gestational sac below vs. above the midline of the uterus (reported by Timor-Tritsch et al.).

The classification system proposed by Kaelin Agten et al. is based on the relationship between the gestational sac and prior cesarean scar: Implantation “on the scar” means that the placenta is implanted partially or fully on top of a well-healed scar (myometrial thickness between the sac and the bladder is ≥ 3mm). In contrast, the implantation “in the niche” means that the placenta is implanted into a deficient or dehiscent scar (myometrium measures ≤2 mm).

There is also the assessment of implantation using “above” vs. “below” the line classification proposed by Timor-Tirsch et al. The diagnosis of CSP (or in the rarest cases, a cervical pregnancy) is determined by the relationship between the gestational sac and the uterine midline (a line drawn perpendicular to the antero-posterior longitudinal axis of the uterus, which divides uterus in half): the center of the gestational sac in CSP is below the half-line, closer to the cervix, as it was the case in our patient. Normal intrauterine gestation is characterized by the center of the sac localized above the half-line, closer to the uterine fundus. Authors of the study concluded that first-trimester diagnosis of the COS-1, pregnancy implantation in the niche, and gestational sac below the uterine midline had high predictive accuracy for the most severe forms of placenta accreta spectrum. All three ultrasound markers were associated independently with adverse surgical outcomes. All mentioned ultrasound signs were encountered at the first transvaginal ultrasound examination of our patient performed during the sixth week.

The apparent larger thickness of the myometrial layer on the ultrasound examination performed during the eighth postmenstrual week (Figure 2) could be explained by the unequal thickness of the myometrial layer over the gestational sac along the cesarean scar and the development of the gestational sac toward the uterine cavity. Therefore, it seems that an ultrasound examination performed earlier in pregnancy is more accurate in predicting the severity of placenta accreta spectrum disorder.

Another ultrasound grading system for cesarean scar pregnancy has been recently developed based on the location of the gestational sac and the amount of myometrium remaining [15]. Grade I CSP is defined as the gestational sac penetrating less than half of the myometrium, whereas grade II CSP is defined as penetration greater than a half the myometrium. In grade III CSP gestational sac develops outside the myometrium. In grade IV CSP, the pregnancy is difficult to identify; the gestational sac is highly vascular. According to the first ultrasound examination (Figure 1), the CSP of our patient could be classified as grade II.

Accurate prediction of the morbidly adherent placenta can be achieved at a 12–16 weeks’ gestation. Ultrasound features suggesting this disorder include non-visible cesarean section scar, bladder wall interruption, thin retroplacental myometrium, presence of intraplacental lacunar spaces, presence of retroplacental arterial-trophoblastic blood flow, and irregular placental vascularization demonstrated by three-dimensional power Doppler [16].

A systematic review of prenatal ultrasound imaging and grading of villous invasiveness reported that the most common signs in placenta accreta spectrum disorders include loss of clear zone (62.1%) and the presence of bridging vessels in placenta accreta, loss of clear zone (84.6%) and subplacental hypervascularity (60%) in placenta increta; placental lacune (82.4%) and subplacental hypervascularity (54.5%) in placenta percreta. None of the mentioned ultrasound signs nor a combination were specific for the depth of accrete placentation [17].

Magnetic resonance imaging (MRI) has high predictive accuracy in assessing the depth and topography of placental invasion [18].

The diagnostic value of ultrasound imaging and MRI in detecting the placenta accreta spectrum is similar [19].

Placenta accreta spectrum disorders are relatively rare, so improvement in detection requires specific centers to develop their expertise. Therefore, it was suggested that such patients should be referred to as tertiary units [20]. Standardized protocol and additional training in detecting the ultrasound signs associated with placenta accreta spectrum are also important to improve the diagnostic accuracy and allow the early diagnosis of CSP and PAS disorders [21].

Clinically, CSP can be diagnosed via routine sonography during early pregnancy in patients with previous cesarean deliveries or may present as an acute emergency with vaginal bleeding or intraabdominal hemorrhage, due to uterine rupture [22].

Two main differential diagnosis that should be considered during the ultrasound examination of the patient with a CSP are cervical pregnancy (more likely to occur in women with no history of cesarean delivery, characterized by typical clinical findings) and spontaneous miscarriage in progress (there is no live embryo or fetus in spontaneous miscarriage and heartbeat cannot be documented). 

The treatment options for CSPs can be medical, surgical (radical and conservative—preserving future fertility) or involve expectant management. Intragestational-sac injection with ultrasound-guidance has the lowest rate of complications—about 10%. Local injections are performed without general anesthesia; they appear to be the most effective intervention and may be specially indicated when future fertility is desired [23]. 

Systemic methotrexate (MTX) and/or the local injection of MTX or potassium chloride have an overall success rate of 62% [24]. Another option is the transvaginal ultrasound-guided injection of absolute ethanol around the gestational sac as a novel method with good clinical effect [25].

Inserting a Foley balloon catheter at the site of CSP can be used in early pregnancy (5–7 weeks) to stop the evolution of the pregnancy by placing pressure on a gestational sac. It was suggested the French-12 size 10-mL silicone balloon catheter, which could be inserted using real-time transabdominal sonographic guidance when the patient has a comfortably full bladder. Transvaginal sonographic guidance could be used after the initial placement to allow more precise placement and assessing the pressure avoiding overinflation of the balloon. A catheter should be kept in place for the next 24 to 48 h, fastened to the patient’s thigh, with antibiotic coverage [26]. 

Suction aspiration or D&C, alone or in combination, for an early cesarean scar pregnancy, are associated with risk of bleeding, with a mean complication rate of about 62%. In the cases of bleeding after the curettage, Foley balloon tamponade is useful to treat the bleeding [25]. 

However, a combination of D&C and suction aspiration with uterine artery embolization lowered the risk of bleeding to 4% of cases [24].

Uterine artery embolization has a complication rate of 47%, and the results are better in combination with another noninvasive treatment [23].

Resection of the gestational sac during laparotomy or using a laparoscopic or transvaginal approach was successful with a high success rate (≥96%) and a low risk of hemorrhage (≤4%). Hysteroscopic resection of CSP was reported to be unsuccessful in 12% of cases, due to inadequate HCG decay, which was the main indication for reintervention [24]. There is also the possibility to combine several treatments.

All aforementioned treatments are associated with complications. The literature supports an interventional rather than a medical approach. However, multicenter, well-designed studies are needed for definitive conclusions regarding the treatment of CSP [27].

On the other hand, some patients with CSP decline termination of their pregnancy. It was recommended that in women who choose expectant management, cesarean delivery should be performed between 34 0/7 and 35 6/7 weeks of gestation [28]. Our patient was asymptomatic; therefore, the decision was made to perform cesarean delivery at 38th week.

Expectant management results in a 57% live birth rate, but the possible complications include uterine rupture with life-threatening hemorrhage, fetal demise, and preterm delivery with neonatal complications [28]. A systematic review of 63 studies revealed that hysterectomy, due to morbidly adherent placenta was performed in 63% of CSP patients who had chosen expectant management [24].

One of the most important studies on CSP reported that four out of ten (40%) CSP patients managed expectantly delivered alive offspring via subsequent elective cesarean deliveries, but three (30%) had hysterectomies for placenta percreta after the delivery [6]. This was also the case with our patient, who had already had a child and decided to take the risk of a possible hysterectomy.

Another problem is uterine rupture and hemorrhage during the second trimester. Timor-Tritsch et al. reported that 5/10 patients with expectant management had second-trimester complications, all leading to hysterectomy [6]. 

A systematic review and meta-analysis, including 17 studies on expectant management of CSP published after 2000 (including 69 cases of CSP managed expectantly, 52 with and 17 without embryonic/fetal heartbeat), reported that 13% patients with embryonic/fetal heart activity experienced an uncomplicated miscarriage, 20.0% required medical or surgical intervention, while uterine rupture during the first and second trimester of pregnancy occurred in 9.9%, and hysterectomy was required in 15.2% during the first and second trimester of pregnancy. Among the women with CSP and embryonic/fetal heart activity, 40 (76.9%) progressed to the third trimester of pregnancy, 39.2% of them had severe bleeding during the third trimester, and uterine rupture occurred in 10.2%. Hysterectomy during cesarean delivery was required in 60.6% of cases. There were no cases of maternal death [29].

It was reported that 69% of CSP patients without detectable embryonic/fetal activity had uncomplicated miscarriage, surgical or medical intervention was required in 31% during or immediately after the miscarriage. The risk for uterine rupture and hysterectomy during the first trimester was negligible in such patients [27].

A recent systematic review and meta-analysis, including forty-four studies (3598 women with CSP), reported that CSP recurred in 17.6% of all women treated for previous CSP, and 82.6% of women had an intrauterine pregnancy. Pregnancy was achieved in 70.6% of cases among women who wished to conceive after a prior CSP. According to the type of management (surgical vs. non-surgical), the pregnancy was achieved in 74.4% and 68.7%, respectively. Women with a prior CSP were at high risk of miscarriage, preterm birth, and placenta accreta spectrum. Subgroup analysis, according to the management of CSP (surgical vs. non-surgical), showed that CSP recurrence rate was 21% and 15.2%, respectively. Miscarriage, preterm birth, and placenta accreta spectrum disorders complicate 16.2%, 8.9%, and 2.7% of pregnancies achieved after the surgical treatment compared with 14.7%, 15.2%, and 10.6% of pregnancies achieved after non-surgical treatment of the prior CSP, respectively. Authors concluded that further prospective studies sharing an objective protocol of prenatal management were needed to evaluate subsequent reproductive outcomes among women with a prior cesarean scar pregnancy (CSP) [30]. It has been already recommended to inform women with a cesarean scar pregnancy about the risks of another pregnancy and counsel them regarding contraception, including permanent contraception [28].

Clearly, CSP represents a great risk for both the mother and the child. On the other hand, there are many mothers who would take such a risk in the presence of embryonic heart activity. The problem is how to determine which patients are candidates for expectant management. Logically, a larger number of previous cesarean deliveries would increase the risk. The thickness of the myometrial layer between the gestational sac and bladder and the progression of pregnancy toward the uterine cavity or outside the uterine cavity are crucial prognostic factors. In our patient, the myometrial layer between the gestational sac and bladder was thinner than on the other part of the anterior uterine wall, but still present. Furthermore, on the ultrasound performed after the eighth postmenstrual week, it was obvious that pregnancy developed towards the uterine cavity, allowing normal development of the embryo. These findings and the possibility of close surveillance under hospital conditions encouraged us to proceed with expectant management. The rest of the pregnancy was uneventful, despite the placenta previa being diagnosed, allowing the patient to reach the 38th week. For these reasons, we consider that definitive decisions on the course of treatment in stable patients who would like to keep their pregnancies should be made after the eighth week. The patient should be informed of the possible obstetric complication. We also suggest that in asymptomatic cases of CSP treated expectantly, the cesarean section should be carried out by an appropriately experienced operator, provided that the patient is hospitalized in a tertiary unit, and a senior anesthetist and other consultants are available at every moment. An intensive care unit and a hospital transfusion laboratory capable of obtaining blood products immediately are required as well. 

The case of our patient showed that it is possible to wait as far as the patient is asymptomatic, so we suggest that in asymptomatic patients, the cesarean delivery should be performed at 38th week. 

Nevertheless, adequate studies on this problem with a substantial number of cases are still lacking [29].

## 4. Conclusions

The early diagnosis of cesarean scar pregnancy is crucial, and adequate treatment can prevent life-threatening complications if the termination of the pregnancy is chosen. Patients who engage in expectant management are at risk of severe complications. Therefore, a close follow-up is mandatory if expectant management is selected. Further studies are needed for definitive conclusions and to determine the risks of expectant management.
